# Stimulating Tourist Inspiration by Tourist Experience: The Moderating Role of Destination Familiarity

**DOI:** 10.3389/fpsyg.2022.895136

**Published:** 2022-07-01

**Authors:** Jianping Xue, Zhimin Zhou, Salman Majeed, Ruixia Chen, Nan Zhou

**Affiliations:** ^1^College of Management, Shenzhen University, Shenzhen, China; ^2^School of Economics and Management, Yibin University, Yibin, China; ^3^International Center for Hospitality Research & Development, Dedman College of Hospitality, Florida State University, Tallahassee, FL, United States; ^4^School of Tourism Management, Henan Finance University, Zhengzhou, China

**Keywords:** tourist inspiration, tourist experience, destination familiarity, inspired-by state, inspired-to state, structural equation model, destination marketing

## Abstract

The tourist experience is a core indicator of destination management for the comprehensive evaluation of destination value. Tourist experience and tourist inspiration are important concepts in the stream of research on destination marketing and management. However, these relationships remained under-explored in the extant literature. This study examined the impact of tourist experience on tourist inspiration under the moderating impact of destination familiarity. To achieve the objective of this study, data were collected online from 622 Chinese tourists. We employed partial least squares structural equation modeling (PLS-SEM) to statistically analyze the gathered data. Findings show that four types of tourist experiences, namely education, esthetics, entertainment, and escapism, significantly and positively influenced the inspired-by state of tourist inspiration, which further influenced the inspired-to-state of tourist inspiration. Destination familiarity exerted a significantly negative moderating impact on the relationship between education experience and inspired-by state of tourist inspiration. Sensitivity analysis presents that education experience was the strongest predictor of the inspired-by state followed by aesthetics, escapism, and entertainment facets of the tourist experience. Findings contribute to the theory and practice of tourism management with a robust interpretation of tourist experience, tourist inspiration, and destination familiarity to solidify the effective management of tourist destinations. Limitations and future research directions are noted.

## Introduction

Shopping and consumption at a tourist destination is an important tourist behavior, which fuels destination revenue and revitalizes local economies, especially in the context of declining tourist inflow during destination crises, such as the COVID-19 pandemic (Rasoolimanesh et al., 2021). Tourist behavior, such as purchase and consumption of destination products and services, is often sudden, temporary, unplanned, and is inspired by destination situation and tourist experience during tourist visit to a destination (Woodside and King, 2001). For example, at the Lantern Festival in China, tourists in Zigong, a city in Sichuan Province of China, get inspired by Zigong’s special cultural lanterns, and make unplanned and sudden purchase decisions about Zigong’s specialty lanterns. Inspiration is a motivational state that promotes the transition from consumption ideas to consumption behavior (Böttger et al., 2017). The inspiration theory advocates that an individual may get inspired by new things to generate novel ideas, and the transcendence of these ideas may promote an individual’s intrinsic motivation to realize new ideas (Thrash and Elliot, 2003).

Tourist inspiration is defined as a motivational state that drives tourists to realize consumption-related new ideas (Böttger et al., 2017; Dai et al., 2022). Inspiration includes three core characteristics, i.e., evocation, transcendence, and approach motivation, and two states, i.e., an inspired-by state and an inspired-to state (Thrash and Elliot, 2004). Evocation means that external stimuli provoke inspiration, which is a spontaneous process rather than self-awakening (Böttger et al., 2017; Dai et al., 2022). Transcendence means that an individual discovers new and better possibilities, which have never been discovered in the past (Winterich et al., 2019), with a feeling of positivity, clarity, and self-enhancement (Böttger et al., 2017). Finally, approach motivation refers to an individual’s inner drive to turn new ideas into actions (Dai et al., 2022). The inspired-by state of tourist inspiration is an epistemic activation component (Böttger et al., 2017) that demonstrates how external stimuli, such as destination environment, activities, and marketing efforts, induce consumption-related ideas into tourist cognitive filters and generate tourist awareness about new and better consumption possibilities (Winterich et al., 2019). The inspired-to state is an intention component (Böttger et al., 2017) that demonstrates how a tourist derives an intrinsic motivation to actualize consumption-related ideas. Tourist inspiration drives consumption-related ideas into tourist consumption behavior, which may provide a potential shortcut to characterize tourist purchase decisions (Dai et al., 2022). Scholars document that the inspired-by-state significantly and positively affects the inspired-to state (Böttger et al., 2017; Izogo et al., 2020). Tourist inspiration occurs when the inspired-by state and the inspired-to-state exist in a causal and sequential manner (Thrash et al., 2014). Previous studies on the tourist decision-making process mainly focused on pre-travel tourist destination choice decisions and ignored tourist consumption and purchase decisions during a tourist visit to the destination (Dai et al., 2022). Research on tourist inspiration and associated consumption-related decision-making process during tourist visit to a destination remained mixed and fragmented in the extant literature, which demands research attention for conclusive evidence.

Tourists are consumers of destination products and services; however, tourist purchase decision might be different from ordinary consumer purchase decision. As a classic theory of the consumer decision process, the Engel-Kollat-Blackwell (EKB) model assumes that consumer decision-making is a completely rational process (McCabe et al., 2016) that passes through five stages, i.e., need recognition, information search, evaluation of alternatives, purchase choice, and post-purchase (Darley et al., 2010). Given the facts of information overload or information insufficiency, cognitive limitations, and personal energy/time/cost constraints, tourists are not fully rational to find and evaluate available potential alternatives (Wattanacharoensil and La-ornual, 2019). Tourist consumption behavior reflects the pursuit of pleasure rather than utility maximization (Dai et al., 2022). The five stages of the EKB model sometimes do not fully correspond to tourist purchase decision-making process. It is because the EKB model’s decision-making stages may be simplified or even omitted by tourists during the information search and evaluation of alternatives during the consumption-related decision-making process. Thus, the EKB model may fail sometimes in explaining tourist consumption-related decision-making processes during tourist visit to destinations. Dual-system theories propose that there are two distinct and complementary decision-making systems. System 1 relies on emotions to make decisions, which is an intuitive response, with rapid, heuristic, and affect-driven characteristics. System 2 relies on cognition to make decisions, which is a process of deliberate considerations, with slow, rational, analytic, and reflective characteristics (Kahneman, 2011). Ordinary consumers who make purchase decisions following the decision-making steps of the EKB model are more likely to rely on System 2 of the dual-system theories, while tourists who make purchase decisions following tourist inspiration are more likely to rely on System 1 of the dual-system theories. Tourists get inspired by discoveries or new experiences obtained from the destination environment, activities, and marketing efforts, which prompt tourists to get new consumption-related ideas (Böttger et al., 2017; Dai et al., 2022). A unique and novel travel-related experience gleaned from tourist interaction with a destination stimulates tourist imagination and fuels tourist inspiration. Since tourist inspiration promotes the transition of new consumption-related ideas to consumption behavior (Böttger et al., 2017), it is important to explore the factors that trigger tourist inspiration, which remained under-explored in the previous studies (Khoi et al., 2019; He et al., 2021). A profound understanding of tourist inspiration may bridge the gap where the EKB theory may not fully explain tourist’s consumption-related decision-making processes during visits to tourist destination.

The tourist experience is defined as an interaction between a tourist and a destination (Stamboulis and Skayannis, 2003). An individual’s experience of destination events may be unique and completely different from that of others (Pine and Gilmore, 1998; Stamboulis and Skayannis, 2003). Scholars document the notion of the tourist experience in terms of education, esthetics, entertainment, and escapism realms of the tourist experience (Oh et al., 2007). The experience economy creates memorable events for individuals (Pine and Gilmore, 1999). From the perspective of the experience economy, the notion of tourist experience has been examined by scholars across different fields (Larsen, 2007; Ritchie and Hudson, 2009; Volo, 2009). Existing research in the stream of tourism attempted to explore the outcomes of tourist experience, such as tourist wellbeing (Hwang and Lee, 2019), pleasant arousal (Loureiro, 2014), and tourist inspiration (He et al., 2021). A study on wellness tourism experience explored the relationship between tourist experience and tourist inspiration and found that education, esthetics, and escapism facets of tourist experience significantly impact tourist inspiration except for entertainment experience (He et al., 2021). Different tourism-related studies emphasize the dimensions of tourist experience differently and report mixed findings in a specific context that limit the generalizability of the findings of the studies. For example, Luo et al. (2018) focused on escaping and education dimensions of the tourist experience in the context of wellness tourism and Choi and Choi (2018) examined the entertainment and escaping dimensions of the tourist experience in the context of mass tourism. He et al. (2021) focused on tourist experience and measured tourist inspiration in the context of wellness tourism. Nevertheless, there is a need to investigate the impact of tourist experience on tourist consumption-related inspiration in the broader context of tourism, such as conventional tourism. This study examines the relationship between tourist experience and tourist consumption-related inspiration in a conventional context of tourism, which is broad, to expand the generalizability of the conceptual understanding of the tourist experience and tourist inspiration. Moreover, this study explores how education, entertainment, esthetics, and escapism dimensions of the tourist experience affect tourist inspiration, i.e., the inspired-by-state that ultimately affects the inspired-to-state of tourist inspiration.

Destination familiarity means tourists’ subjective assessment of their existing knowledge and information alongside learning new knowledge and skills during visits to tourist destinations (Hernández Maestro et al., 2007). Some tourists prefer to choose unfamiliar destinations to obtain a novel travel experience (Chark et al., 2020), which increases the probability of tourist inspiration, while some tourists get inspired by familiar destinations to obtain a stable travel experience with intentions to reduce travel-related uncertainties, which reduces the probability of tourist inspiration (Kim et al., 2019). From this, it is discerned that destination familiarity exerts its impact on the relationship between tourist experience and tourist inspiration. Existing studies show that tourists who are familiar with a destination exhibit positive attitude and behavioral intentions, such as intention to visit tourist destination (Chaulagain et al., 2019; Shi et al., 2022), satisfaction (Sanz-Blas et al., 2019), and destination evaluation (Chen et al., 2017; Kim et al., 2019). Scholars believe that destination familiarity may increase tourist confidence in choosing a destination in parallel to triggering tourist decision-making process to choose familiar destinations (Milman and Pizam, 1995). Tourists’ prior knowledge or destination familiarity may increase tourists’ sense of safety and reduce tourists’ perceptions of perceived risk during visits to destinations (Karl, 2016).

An individual’s familiarity affects his/her information search behavior (Gursoy, 2019). While taking decisions to visit a tourist destination and consume destination products and services, tourists who are familiar with destinations reduce their search for external information regarding tourist destination because such familiar tourists hold sufficient destination information (Gursoy and McCleary, 2004a). On the other hand, unfamiliar tourists lack sufficient destination-related information and, thus, increase their search for external information regarding tourist destinations to reduce the levels of uncertainty and perceived risk during travel to tourist destinations (Carneiro and Crompton, 2009; Karl, 2016). The inspiration theory maintains that inspiration is triggered by external stimuli rather than internal self-awakening (Böttger et al., 2017; Dai et al., 2022). External stimuli may likely trigger more tourist inspiration in low-familiar tourists as compared to high-familiar tourists (Böttger et al., 2017; Winterich et al., 2019). From the perspective of tourist consumption-related decision-making, tourists who are familiar with a destination are more likely to make decisions based on the system 2 approach of the dual-system theories, which is similar to the theoretical premises of the EKB model. It is because high-familiar tourists might have sufficient destination information for a rational decision to visit a tourist destination (McCabe et al., 2016). However, low-familiar tourists are more likely to make decisions based on the system 1 approach of the dual-system theories, which is similar to tourist consumption-related inspiration. It is because tourists might lack sufficient destination information and rely on their intuition and cognitive reactions to external stimuli for consumption-related destination decision-making (McCabe et al., 2016). However, studies that demonstrate the role of tourist destination familiarity in tourist decision-making to visit tourist destinations remained succinct. Drawing on the above and to bridge the identified research gap, this study also examines how destination familiarity exerts its moderating impact on the relationship between tourist experience and tourist inspiration.

This study aims to examine the following: (1) How does tourist inspired-by state impact tourist inspired-to state? (2) How does tourist experience impact tourist inspiration, i.e., tourist inspired-by state? (3) How does tourist destination familiarity exert its moderating impact on the relationship between tourist experience and tourist inspiration, i.e., tourist inspired-by state? Findings unravel the psychological mechanism of tourist purchase motivation from the perspective of tourist inspiration. This study fills theoretical gaps with a proposed conceptual framework and offers guidelines to destination marketing organizations (DMOs) in solidifying destination management and promotion efforts to skyrocket sales revenue of tourist destination. This study provides roadmaps for scholars and practitioners to conduct future research on destination marketing and management.

## Literature Review

### Tourist Inspiration: Inspired-by State and Inspired-to State

Customer inspiration is defined as a state of temporary motivation evoked by corporate marketing efforts, promoting the generation of new ideas related to consumption, and driving consumers to take action on new ideas (Böttger et al., 2017). As an important concept of the inspiration theory in marketing, customer inspiration includes inspired-by and inspired-to states in its breadth and depth and focuses on the generation of new ideas inspired by corporate marketing efforts and customer consumption behavior (Böttger et al., 2017). Tourist inspiration and its cognitive importance in tourist decision-making have gained the widespread attention of scholars and practitioners from different fields (Khoi et al., 2019; Khoi et al., 2021; Dai et al., 2022). Dai et al. (2022) note the importance of travel inspiration at the tourist dreaming stage and refer travel inspiration as a motivational state that may influence tourist behavior, such as tourist destination choice. Gollwitzer (1990) proposed the mindset theory of action phases and divided the consumer decision-making process into two phases i.e., a pre-decision phase of deliberation and a post-decision phase of implementation, embodying a sequential relationship. Böttger et al. (2017) argue that the inspired-by state belongs to the deliberation phase, while the inspired-to-state reflects the transition to the implementation phase. In the stream of tourist inspiration, there is a causal and sequential relationship between the inspired-by state and the inspired-to state where the inspired-by-state exists before the inspired-to state (Böttger et al., 2017; Cao et al., 2021; Dai et al., 2022).

External stimuli, such as destination environment, destination events, and discovery of new possibilities in a tourist destination, may trigger tourist inspired-by-state in tandem with generating new ideas regarding tourist consumption with self-transcendence (Khoi et al., 2019). According to the theory of self-determination, the attractiveness of new ideas regarding tourist consumption drives tourist self-realization and fuels tourist unplanned purchases (Ryan and Deci, 2000), which is sketched as the inspired-to state of tourist inspiration on the canvas of this study. For example, when a tourist is inspired by a product with destination characteristics, the idea of purchasing such a product and giving the same to a friend as a gift may emerge naturally (Böttger et al., 2017). The appropriateness of the product as a gift becomes an internal driving force that generates tourist purchase intention, which is an inspired-to state of tourist inspiration (Thrash and Elliot, 2004). Some scholars argue that inspired-by state may also be a precursor to inspired-to state (Hinsch et al., 2020; Izogo et al., 2020; Izogo and Mpinganjira, 2020). For example, in a cross-cultural study, Izogo et al. (2020) found that inspired-by state significantly and positively affects the inspired-to state. Similar findings were found in research on augmented reality (Hinsch et al., 2020) and social media content (Izogo and Mpinganjira, 2020). However, most studies advocate the causal and sequential impact of inspired-by state on inspired-to state (Böttger et al., 2017; Dai et al., 2022). Thus, there is a need to testify the impact of inspired-by state on inspired-to state for conclusive evidence. Drawing on most studies reflecting the impact of inspired-by state on inspired-to state, we propose the following hypothesis.

**Hypothesis H1**: Tourist inspired-by state exerts a significantly positive impact on the inspired-to state of tourist inspiration.

### Tourist Experience

The tourist experience is the application of the experience economy in a tourism context. The concept of the experience economy is widely accepted by tourism scholars and practitioners. According to the two dimensions of customer connections, namely absorption and immersion, and the level of customer participation, namely passive and active participation, four experience realms of tourist experience are identified in the extant literature for business promotion, i.e., education, esthetics, entertainment, and escapism (Pine and Gilmore, 1999). A unique tourist experience stems from an interaction between destination event and tourist cognitive reaction (Pine and Gilmore, 1998) that may stimulate tourist psychological arousal (Hosany and Witham, 2010) alongside provoking tourist inspiration (Khoi et al., 2019). Tourist experience influences tourist psychological arousal, which is the first step toward tourist inspiration (Loureiro, 2014).

#### Education Experience

Education experience may impact tourist inspiration (Whiting and Hannam, 2014). Tourists attempt to find new ways to gain new knowledge about tourist destinations to improve consumption-related decision-making (Oh et al., 2007). Education experience has two characteristics, i.e., active participation and absorption (Pine and Gilmore, 1998). From the perspective of tourism, education experience allows tourists to acquire new knowledge and skills to identify new and better possibilities, stimulates tourist imagination, and generates new ideas relevant to tourist consumption of destination products and services (Winterich et al., 2019). Since tourist inspired-by state is an epistemic activation process, which reflects evocation and transcendence of tourist inspiration (Böttger et al., 2017), tourists learn new knowledge and skills as a part of educational experience during visits to a tourist destination that may inspire tourists, trigger tourist novel consumption-related ideas, and grab tourist attention, which reflects the evocation characteristics of tourist inspiration (Pine and Gilmore, 1999; Dai et al., 2022). The discovery of new and better possibilities during visits to a tourist destination allows tourists to realize the quality of new ideas and gain a sense of self-transcendence, which reflects the transcendence characteristics of tourist inspiration (Thrash and Elliot, 2003). Therefore, we propose the following hypothesis.

**Hypothesis H2**: Education experience exerts a significantly positive impact on the inspired-by state of tourist inspiration.

#### Entertainment Experience

Personal experience linked to entertainment is the most emphasized dimension of the tourist experience in destination marketing (Pine and Gilmore, 1999). Destination performances and activities attract tourist attention and make tourists feel happy and excited about destination performances and activities (Oh et al., 2007). During this process, tourists do not directly participate in destination activities, which presents that entertainment experience encapsulates the characteristics of passive participation and absorption in its breadth and depth. It is noted that entertainment experience provokes tourist positive emotions when tourists watch a destination performance (Hwang and Lee, 2019). The broaden-and-build theory in the field of positive psychology argues that positive emotions broaden individuals’ cognitive scope alongside building individuals’ physical, intellectual, social, and psychological resources (Fredrickson, 2001). Existing shreds of evidence demonstrate that the broaden-and-build theory has been applied to research on tourist wellbeing (Sirgy, 2019), value co-creation (Lin et al., 2017), and social media sharing behavior (Chen et al., 2021). Tourists with positive emotions are more open-minded, flexible to adopt changes, and able to generate more creative ideas, which can promote tourist self-efficacy to overcome destination challenges (Chen et al., 2021). According to the broaden-and-build theory, positive emotions instantly expand an individual’s creative thinking (Fredrickson, 2001). Watching destination performances may stimulate tourists’ happy feelings, promote broader and more imaginative tourist thinking, and generate new ideas regarding tourist consumption. Destination performances and activities may activate tourists’ positive emotions to gain new consumption-related ideas, which reflect the evocation characteristic of tourist inspired-by state. New ideas and creative solutions induce a feeling of self-transcendence, which reflects the transcendence characteristic of the inspired-by state of tourist inspiration (Thrash and Elliot, 2003). Thus, we also propose the following hypothesis.

**Hypothesis H3**: Entertainment experience exerts a significantly positive impact on the inspired-by state of tourist inspiration.

#### Esthetics Experience

The esthetics experience is a process in which tourists feel and appreciate objective matters and the environment (Pine and Gilmore, 1999). Tourists feel completely immersed in the objective environment and start perceiving and explaining esthetic meanings of destination environment from their unique perspectives, which may trigger tourist inspiration (Khoi et al., 2019). Hosany and Witham (2010) document that an individual’s interpretation of the physical environment fuels an individual’s esthetic experience. Tourists are immersed in the destination environment, passively appreciate, and feel destination beauty, and do not intend to bring changes to destination environment (Oh et al., 2007). This process requires an individual’s full concentration on the environment (Pine and Gilmore, 1999). Therefore, esthetic experience mirrors the characteristics of both passive participation and immersion (Pine and Gilmore, 1998). Esthetic appreciation has a strong cognitive component that requires tourists to invest energy and cognitive resources. When tourists are inspired by the beauty of objective matters or the environment of a destination, tourists discover new and better possibilities in generating consumption-related new ideas (Khoi et al., 2019). Drawing on the above, we propose the following hypothesis.

**Hypothesis H4**: Esthetics experience exerts a significantly positive impact on the inspired-by state of tourist inspiration.

#### Escapism Experience

Escaping reality is an important motivation for tourists (Kozak, 2002). Tourists temporarily escape the unsatisfactory aspects of daily life and seek places to tour and participate in activities arranged at tourist destinations (Oh et al., 2007). Therefore, escapism has the characteristics of both active participation and immersion (Pine and Gilmore, 1998). To escape reality, tourists expect to travel to specific tourist destinations and participate in destination activities to distance themselves from daily life matters for rest, relaxation, and a feeling of stress alleviation. Traveling to tourist destinations allows tourists to feel that they are in a different time and space and this feeling helps tourists to enjoy a new lifestyle with new and better possibilities of life activities in tourist destinations alongside inspiring tourists with new consumption-related ideas (Pine and Gilmore, 1999). Based on the above, we propose the following hypothesis.

**Hypothesis H5**: The escapism experience exerts a significantly positive impact on the inspired-by state of tourist inspiration.

### The Moderating Role of Destination Familiarity

As an important construct in the field of marketing, familiarity with a product or brand refers to consumer experience and knowledge about a product or brand that may influence consumer decision-making regarding a product or brand (Alba and Hutchinson, 1987). Previous studies present that consumer positive decision-making and resultant favorable behavior are linked to consumer familiarity with brand products or services, such as first-time and repeat purchases (Tam, 2008; Paasovaara et al., 2012), as compared to consumer unfamiliarity with brand products or services. Destination familiarity is defined as an individual’s subjective assessment of destination information and knowledge (Hernández Maestro et al., 2007). Tourists’ behavioral intentions are influenced by tourists’ subjective familiarity assessment of destination attributes (Rao and Sieben, 1992; Park et al., 1994). When tourists feel that they are familiar with a tourist destination, they are more confident in their decision-making to visit tourist destinations (Milman and Pizam, 1995).

Existing studies show that destination familiarity has an important impact on tourist information search behavior (Gursoy, 2019). Tourists first conduct an internal search to obtain desired information from their memory and experience (Coupey et al., 1998). Tourists who extract desired information from their memory attempt to make informed decisions and do not engage in additional information search from external sources (Brucks, 1985). Tourists who do not find the desired information from their memory and experience search for information from external sources for rational travel-related decision-making (Gursoy and McCleary, 2004b). Evocation as a salient feature of tourist inspiration means that tourist inspiration is spontaneously evoked by an external stimulus (Böttger et al., 2017; Dai et al., 2022). Tourist inspiration is more likely to be triggered in low-familiar tourists as compared to in high-familiar tourists, because information obtained from outside may help tourists learn new knowledge, make discoveries, and gain new insights, which stimulate tourist imagination (Winterich et al., 2019). High-familiar tourists extract information from their memory and experience for decision-making, which may not fully trigger tourist inspiration due to the lack of external new things (Böttger et al., 2017). Tourists who are familiar with destinations show the favorable evaluation of destination attributes and develop positive behavioral intentions as compared to unfamiliar destinations (Chaulagain et al., 2019; Sanz-Blas et al., 2019; Shi et al., 2022). Previous studies note that destination familiarity moderates the relationship between brand equity and tourist revisit intention (Shi et al., 2022), the relationship between logotype and tourist attitude toward a destination (Roy and Attri, 2022), and the relationship between perceived quality and tourist visit intention (Chi et al., 2020). As tourist familiarity with a destination will increase, novel experiences and discoveries from external stimulation will decrease, which will reduce tourist inspiration. Therefore, we also propose the following hypotheses.

**Hypothesis H6**: Destination familiarity exerts a significant moderating impact on the relationship between education experience and the inspired-by state of tourist inspiration such that the relationship is weak when destination familiarity is high.
**Hypothesis H7**: Destination familiarity exerts a significant moderating impact on the relationship between entertainment experience and the inspired-by state of tourist inspiration such that the relationship is weak when destination familiarity is high.
**Hypothesis H8**: Destination familiarity exerts a significant moderating impact on the relationship between esthetics experience and the inspired-by state of tourist inspiration such that the relationship is weak when destination familiarity is high.
**Hypothesis H9**: Destination familiarity exerts a significant moderating impact on the relationship between escapism experience and the inspired-by state of tourist inspiration such that the relationship is weak when destination familiarity is high.

**FIGURE 1:**
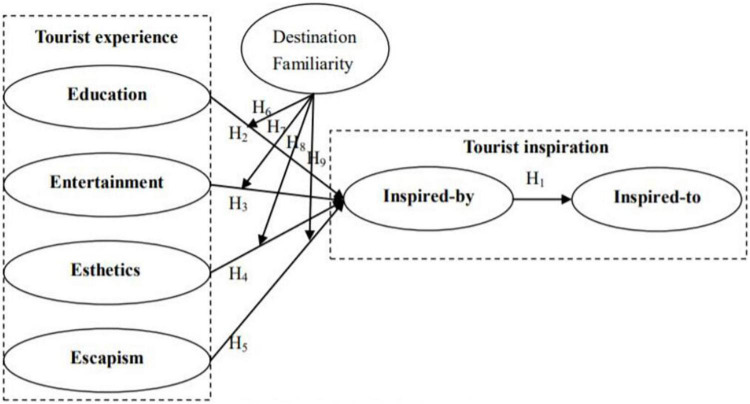
Conceptual model.

The proposed theoretical associations among the constructs of this study are presented in Figure 1.

## Methodology

### Questionnaire Design

A survey questionnaire was designed to gather data from Chinese respondents having tourist experience. Screening questions were made a part of the survey questionnaire: (1) What type of destination was your last trip? (2) What is the name of your recently visited tourist destination? (3) How recent is your tourist experience? To measure the proposed constructs in this study, scale items were adapted from the previous studies. Scale items for education, esthetics, entertainment, and escapism dimensions of tourist experience were adapted from Oh et al. (2007), scale items for tourist inspiration (including the inspired-by state and the inspired-to state) were adapted from Böttger et al. (2017), and scale items for destination familiarity were adapted from Gursoy and McCleary (2004a). All scale items were measured using a seven-point Likert scale, which ranged from 1 (strongly disagree) to 7 (strongly agree) (see Appendix 1). Scholars document that the seven-point Likert scale is well-suited to conduct online surveys (Chaulagain et al., 2019). The survey questionnaire was originally developed in English. Three native Chinese doctoral students, who were proficient in English and had academic and industry experience in tourism marketing, were invited to translate the English version of the questionnaire into Chinese. By following the work of Majeed et al. (2020a), we used the blind-translation back-translation method to translate the English version of the questionnaire into the Chinese language. Two bilingual professors, who were unfamiliar with the field were invited to convert the translated Chinese version of the survey questionnaire into English. The quality of the English and Chinese versions of the survey questionnaires were compared for clarity and similar intended meanings of the questions and minor adjustments were made to the content and composition of the survey questionnaire before the full launch of the survey (Chen et al., 2020).

### Data Collection

This study conducted an online survey at Wenjuan Xing^1^, a professional online academic survey platform in China, between November 5, 2019 and November 10, 2019, to gather data from Chinese tourists who were at least 18 years old to ensure the consent requirement of the study respondents (Majeed et al., 2020b; Xue et al., 2020). Wenjuan Xing undertakes a random sampling method to administer surveys and records responses from its more than three million registered users, who demonstrate diverse backgrounds and belong to different cities in China (Cao et al., 2021). Wenjuan Xing adopts a multichannel approach to distribute questionnaires to randomly invited users to reflect a greater representation of the relevant study population for the survey (Cao et al., 2021). The participants in the online survey were Chinese adult tourists who had traveled to tourist destinations. A total of 626 responses were received during the data collection process. After removing responses carrying missing values and respondent age as less than 18 years, a total of 622 were retained for the final analysis.

### Study Respondents’ Demographic Details

The number of male and female respondents in this study was relatively balanced, accounting for 45.7 and 54.3%, respectively (see Table 1). Most respondents were under the age of 40 years (80.38%), corporate staff (66.1%), and were married (67.2%). Table 1 shows that approximately 67.4% of the study respondents mentioned their education level as undergraduate. Approximately 63.5% of the study respondents mentioned 2–3 trips per year, 62.8% of respondents traveled for the first time, 50.6% of respondents liked to travel with family, and 31.5% respondents liked to travel with friends. Approximately 94.1% of respondents traveled to domestic tourist destinations. Destinations related to natural and heritage landscapes were favored by most of the study respondents, i.e., 47.7 and 42.1%, respectively. Approximately 74.8% of the study respondents visited tourist destinations within the previous 3 months from the date of the survey.

**TABLE 1:** Demographic and destination characteristics.

Characteristics	Frequency	Percentage (%)
**Gender**		
Male	284	45.7
Female	338	54.3
**Age**		
18∼29	259	41.6
30∼39	241	38.7
40∼49	93	15.0
50∼59	27	4.3
60 years and over	2	0.3
**Education**		
High school and below	53	8.5
Junior college	112	18.0
Undergraduate	419	67.4
Graduate and above	38	6.1
**Career**		
Student	65	10.5
Corporate staff	411	66.1
Civil servant	99	15.9
Other	47	7.6
**Marital status**		
Single	194	31.2
Married	418	67.2
Other	10	1.6
**Travel frequency (per year)**		
Once	118	19.0
2∼3 times	395	63.5
>3 times	109	17.5
**Destination region**		
Domestic	585	94.1
International	37	5.9
**Destination type**		
Natural landscape	297	47.7
Heritage landscape	262	42.1
Artificial landscape	63	10.1
**How long ago (months)**		
<3	465	74.8
3∼12	128	20.6
>12	29	4.7
**Travel companion**		
Alone	52	8.4
Family	315	50.6
Friend	196	31.5
Other	59	9.5
**Visit frequency**		
First	393	62.8
Revisit	233	37.2

## Results

### Data Analysis Strategy

The four realms of tourist experience and the two states of tourist inspiration are lower-order latent variables in this study. Partial least square structural equation modeling (PLS-SEM) was employed to test the proposed relationships among tourist experience, tourist inspiration, and destination familiarity. PLS-SEM is preferred to the co-variance-based approach because PLS-SEM is one of the most used methods in analyzing the structural relationships of latent variables (Chin and Newsted, 1999) and moderating roles (Wong, 2016; Nitzl and Chin, 2017). Smart PLS 3.2.7 was used for data analysis in this study.

### Measurement Model Results

Table 2 presents the items and reliability evaluation results of the constructs of the study. Except for three items in the range from 0.667 to 0.697, which is above the minimum acceptable value of 0.50 recommended for factor loadings (Hair et al., 2010), all factor loadings were above 0.70 and were considered acceptable (Chin, 1998). The Cronbach’s alpha and composite reliability values of all constructs were above the recommended threshold of 0.70 (Nunnally and Bernstein, 1994), indicating good internal reliability of the study constructs.

**TABLE 2:** Measurement model results.

Constructs and items	Factor loadings
**Education (α = 0.801; CR = 0.867; AVE = 0.621)**	
This tour has expanded my knowledge.	0.786
This tour allowed me to learn a lot.	0.832
This tour inspired my curiosity about learning new things.	0.736
This tour was a learning experience.	0.795
**Esthetics (α = 0.791; CR = 0.849; AVE = 0.586)**	
This tour made me feel harmony.	0.782
This tour was extremely pleasant.	0.78
The surroundings of tourist destination attractions were pretty bland*^a^*.	0.667
The destination environment was very attractive.	0.824
**Entertainment (α = 0.852; CR = 0.900; AVE = 0.693)**	
Activities at tourist destinations were amusing to watch.	0.828
The performance at tourist destination was captivating to watch.	0.831
I enjoyed watching the performance at tourist destination.	0.838
Activities at tourist destination were fun to watch.	0.834
**Escapism (α = 0.843; CR = 0.886; AVE = 0.662)**	
I feel different on this tour.	0.842
This trip made me feel like living in another space and time.	0.845
This trip made me imagine being another self.	0.877
This trip allowed me to escape from reality.	0.677
**Inspired-by (α = 0.816; CR = 0.874; AVE = 0.582)**	
This tour activated my imagination.	0.792
A new idea on this tour caught my interest.	0.786
This trip unexpectedly and spontaneously gave me new ideas.	0.802
This trip has broadened my horizons.	0.732
This tour made me discover something new.	0.697
**Inspired-to (α = 0.880; CR = 0.944; AVE = 0.772)**	
This tour inspired me to buy something related to tourist attractions.	0.865
This trip gave me a desire to buy something related to tourist attractions.	0.889
This tour increased my interest in buying something related to tourist attractions.	0.883
This trip motivated me to buy something related to tourist attractions.	0.882
This trip gave me the urge to buy something related to tourist attractions.	0.874
**Destination familiarity (α = 0.869; CR = 0.886; AVE = 0.722)**	
Before going to tourist attractions, I had more knowledge of tourist attractions than average people.	0.868
Before going to tourist attractions, I had more knowledge of tourist attractions than my friends.	0.872
Before going to tourist attractions, I had more knowledge of tourist attractions than people who travel frequently.	0.807

*AVE, average variance extracted; CR, composite reliability; α, Cronbach’s alpha.*

*^a^Reverse coded.*

The convergent validity and discriminant validity of each construct were evaluated. The average variance extracted (AVE) of all constructs ranged from 0.582 to 0.772 (see Table 2), which is above the recommended threshold of 0.50, indicating that all constructs of the model have good convergent validity (Fornell and Larcker, 1981). The Fornell-Larcker criterion and the heterotrait-monotrait (HTMT) ratio were calculated to evaluate the discriminant validity of the constructs (Henseler et al., 2015; Hair et al., 2021). Table 3 shows that the square root of the AVE of each construct was found greater than the correlation coefficient of other constructs, indicating that all constructs have good discriminant validity and met the Fornell-Larcker criterion. The maximum value of the HTMT ratio (Table 4) was found as 0.857, which is less than the recommended threshold of 0.90 (Henseler et al., 2015; Rasoolimanesh et al., 2021), indicating that all constructs had acceptable discriminant validity.

**TABLE 3:** Fornell-Larcker criterion.

	1	2	3	4	5	6	7
1 Education	***0.788***						
2 Entertainment	0.514	***0.833***					
3 Escapism	0.455	0.404	***0.814***				
4 Esthetics	0.506	0.567	0.430	***0.765***			
5 Destination familiarity	0.314	0.302	0.369	0.263	***0.850***		
6 Inspired-by	0.691	0.589	0.561	0.599	0.295	***0.763***	
7 Inspired-to	0.297	0.438	0.336	0.344	0.168	0.458	***0.879***

*Bold diagonal values represent the square root of AVEs.*

**TABLE 4:** Heterotrait-monotrait (HTMT) ratio.

	1	2	3	4	5	6	7
1 Education							
2 Entertainment	0.621						
3 Escapism	0.542	0.467					
4 Esthetics	0.641	0.688	0.528				
5 Destination familiarity	0.381	0.357	0.441	0.322			
6 Inspired-by	0.857	0.701	0.670	0.747	0.353		
7 Inspired-to	0.345	0.491	0.387	0.405	0.191	0.522	

### Structural Model Results

**FIGURE 2:**
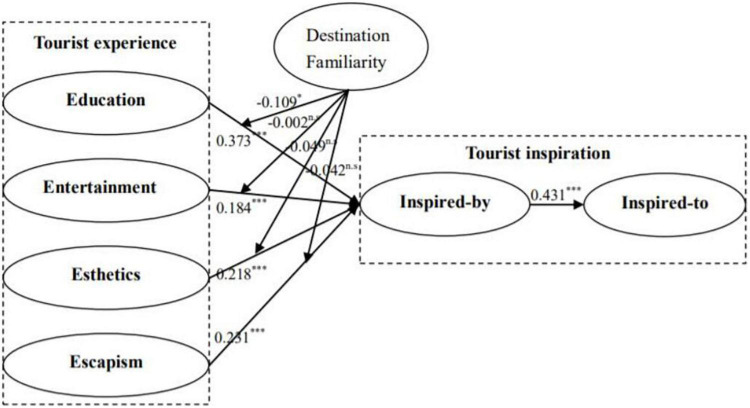
Path co-efficient model. *P < 0.05, ^**^P < 0.01, ^***^P < 0.001, *^n.s^P* > 0.05.

Before the bootstrapping procedure, destination familiarity was set as a moderator, inspired-by state as a dependent variable, and education, entertainment, esthetics, and escapism dimensions of tourist experience as independent variables to generate four moderating impacts. Since destination familiarity is a reflective-reflective construct in this study, a product indicator calculation method was selected while generating the moderating impact on the relationship between dimensions of tourist experience and the inspired-by state of tourist inspiration. To exclude the influence of other factors, demographic and destination variables mentioned in Table 1 were operationalized in the model as control variables. Complete bootstrapping with 5,000 sub-samples was performed to examine the hypothesized relationships presented in the conceptual model. Findings (Table 5) show that *R*^2^ values of the dependent variables are over 0.1 and *Q*^2^ values are over 0, presenting the predictive ability and predictive relevance of the structural model (Falk and Miller, 1992). To solidify the investigation of the goodness of fit and the significance of hypothesized relationships in the model, path coefficients were examined. Findings (Table 5) show that inspired-by state has significantly positive impact on inspired-to state (β = 0.431, *t* = 10.456, *p* < 0.001). Thus, hypothesis H1 is supported. Findings for education experience (β = 0.373, *t* = 9.606, *p* < 0.001), entertainment experience (β = 0.184, *t* = 4.685, *p* < 0.001), esthetics experience (β = 0.218, *t* = 5.612, *p* < 0.001), and escapism experience (β = 0.231, *t* = 7.122, *p* < 0.001) show that education, entertainment, esthetics, and escapism dimensions of tourist experience exert significantly positive impact on the inspired-by state of tourist inspiration. Therefore, hypotheses H2, H3, H4, and H5 are supported. Findings show that destination familiarity has a significantly negative impact on the relationship between education experience and inspired-by state of tourist inspiration (β = −0.109, *t* = 3.095, *p* < 0.05), indicating that when tourist destination familiarity is high, the relationship between education experience and the inspired-by state of tourist inspiration is weak. This supports hypothesis H6. However, findings show that destination familiarity has no moderating impact on the relationship between entertainment experience and inspired-by state (β = 0.002, *t* = 0.051, *p* > 0.05), between esthetics experience and inspired-by state (β = 0.049, *t* = 1.523, *p* > 0.05), and between escapism experience and inspired-by state (β = 0.042, *t* = 1.38, *p* > 0.05). Thus, hypotheses H7, H8, and H9 are not supported. The structural model of the study is presented in Figure 2.

**TABLE 5:** Path analysis.

Hypotheses	Path relationship	Coefficients/β	*t*-value	Results
H1	Inspired-by -> Inspired-to	0.431***	10.456	Supported
H2	Education -> Inspired-by	0.373***	9.606	Supported
H3	Entertainment -> Inspired-by	0.184***	4.685	Supported
H4	Esthetics -> Inspired-by	0.218***	5.612	Supported
H5	Escapism -> Inspired-by	0.231***	7.122	Supported
H6	Education * Destination familiarity -> Inspired-by	−0.109*	3.095	Supported
H7	Entertainment * Destination familiarity -> Inspired-by	0.002	0.051	Not supported
H8	Esthetics * Destination familiarity ->Inspired-by	0.049	1.523	Not supported
H9	Escapism * Destination familiarity -> Inspired-by	0.042	1.38	Not supported
	*R*^2^ inspired-by = 0.201	*Q*^2^ inspired-by = 0.110		
	*R*^2^ inspired-to = 0.199	*Q*^2^ inspired-to = 0.126		

*Destination familiarity = the moderating role of destination familiarity; *p < 0.05, ***p < 0.001.

### Sensitivity Analysis

The objective of sensitivity analysis is to determine how much of the change in the dependent variable is caused by the change in the relevant independent variable (Han et al., 2021) and to obtain a ranking of the importance of the independent variable’s influence on the dependent variable (Ahani et al., 2017; Leong et al., 2020). Artificial neural network (ANN) modeling is the most used method for sensitivity analysis because of its obvious advantages over traditional statistical methods (Sharma et al., 2021), such as regression analysis. ANN does not require the data to follow a normal distribution and is also suitable for the analysis of non-linear relationship variables (Tan et al., 2014; Leong et al., 2020). An ANN model usually consists of three layers, namely the input layer, the hidden layer, and the output layer, and each layer is connected by an activation function (Sharma et al., 2021). In terms of activation function, the sigmoid function is generally considered by researchers due to its advantages of squeezing the original data (Chiang et al., 2006). The IBM’s SPSS 21 neural network module and its multilayer perceptron were employed to perform ANN analysis and a feed-forward-backward-propagation (FFBP) algorithm for training and testing data in this study (Taneja and Arora, 2019). In line with Leong et al. (2020), 90% of data was used for training, while the remaining 10% of data was used for testing where sigmoid is the activation function for the hidden and output layers (Sharma and Sharma, 2019). Referring to the method of Sharma et al. (2021), a 10-fold cross-validating procedure was used to avoid the overfitting problem in ANN analysis. The root mean square error (RMSE) is widely used by scholars to validate the results of the ANN analysis (Chong, 2013; Liébana-Cabanillas et al., 2017), and, hence, followed for ANN analysis in this study.

**FIGURE 3:**
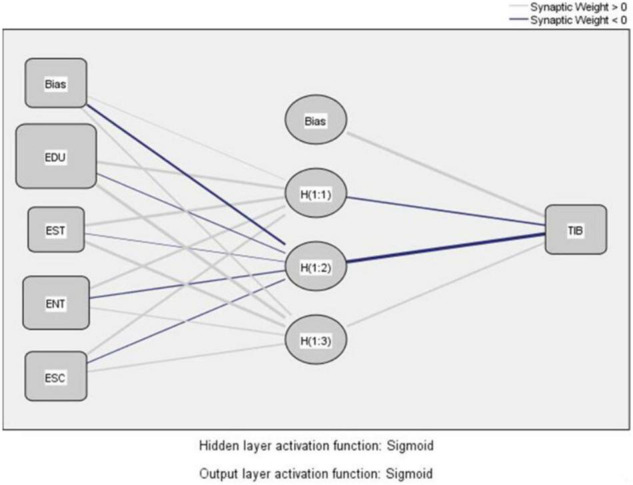
Artificial neural network architecture.

To analyze the importance of education, entertainment, esthetics, and escapism dimensions of tourist experience and their associated impact on the inspired-by state of tourist inspiration, we constructed one ANN model. In the ANN model, the four dimensions of tourist experience, i.e., education, entertainment, esthetics, and escapism, are in the input layer, the inspired-by state of tourist inspiration is in the output layer, and there are three hidden nodes in the hidden layer (see Figure 3). Table 6 shows that the average RMSE values for both training and testing processes were relatively small at 0.074, indicating an excellent model fit (Leong et al., 2019; Leong et al., 2020). To rank the predictive power of the input neurons, a sensitivity analysis was performed. Table 7 shows the importance and normalized importance of each input neuron, i.e., education, esthetics, entertainment, and escapism dimensions of the tourist experience. The value of normalized importance refers to the importance of each input neuron divided by the maximum importance and expressed as a percentage (Leong et al., 2020). The results of the sensitivity analysis showed that education experience had the greatest normalized importance at 98.1%, suggesting that education experience was the most powerful predictor of the inspired-by state followed by esthetics (59.3%), escapism (46%), and entertainment (37.7%) dimensions of the tourist experience.

**TABLE 6:** Root mean square error (RMSE) for artificial neural network model.

ANN	Training			Testing				
			
	N	SSE	RMSE	N	SSE	RMSE	Total sample
ANN01	555	2.891	0.072	67	0.333	0.070	622
ANN02	546	2.751	0.071	76	0.563	0.086	622
ANN03	563	3.039	0.073	59	0.251	0.065	622
ANN04	557	2.904	0.072	65	0.418	0.080	622
ANN05	567	2.921	0.072	55	0.402	0.085	622
ANN06	558	2.962	0.073	64	0.221	0.059	622
ANN07	554	2.859	0.072	68	0.299	0.066	622
ANN08	558	3.114	0.075	64	0.322	0.071	622
ANN09	562	4.509	0.090	60	0.389	0.081	622
ANN10	558	2.881	0.072	64	0.356	0.075	622
Mean		3.083	0.074		0.355	0.074	
Standard deviation		0.511	0.006		0.097	0.009	

*SSE, sum square of errors; N, sample size; ANN, Artificial neural network model.*

**TABLE 7:** Sensitivity analysis.

ANN	EDU	EST	ENT	ESC
ANN01	0.425	0.221	0.155	0.199
ANN02	0.407	0.193	0.192	0.209
ANN03	0.436	0.208	0.174	0.183
ANN04	0.413	0.266	0.124	0.197
ANN05	0.435	0.242	0.107	0.216
ANN06	0.424	0.192	0.188	0.196
ANN07	0.385	0.271	0.161	0.183
ANN08	0.401	0.297	0.128	0.174
ANN09	0.347	0.430	0.074	0.150
ANN10	0.400	0.145	0.253	0.202
Average importance	0.407	0.246	0.156	0.191
Normalized importance	98.1%	59.3%	37.7%	46.0%

*EDU, education; EST, esthetics; ENT, entertainment; ESC, escapism; ANN, Artificial neural network model.*

## Discussion and Conclusion

The findings of this study show that tourist inspired-by state significantly and positively influenced tourist inspired-to state which is in line with the previous studies demonstrating a significant and positive correlation between the inspired by state and the inspired-to state of customer inspiration (Böttger et al., 2017; Hinsch et al., 2020; Izogo et al., 2020). For example, Böttger et al. (2017) found that inspirational content affects the inspired-to state through the inspired-by state in the high idea shopping condition. Hinsch et al. (2020) and Izogo et al. (2020) also confirmed the significant and positive relationship between tourist inspired-by state and tourist inspired-to state in the context of research on cross-culture and augmented reality topics that support the findings of this study. Mediation analysis (see Appendix 2) shows the significant mediating role of inspired-by state between the relationships of education, entertainment, esthetics, and escapism dimensions of the tourist experience and inspired-to state of tourist inspiration. Since the direct impact of inspired-by state on inspired-to state is significantly positive, the significant mediating role of tourist inspired-by state between the dimensions of the tourist experience and inspired-to state of tourist inspiration reflects partial mediation and solidifies the existance of a causal relationship between inspired-by and inspired-to states of tourist inspiration. As stated in the theory of self-determination, the transition of tourist inspiration from the inspired-by state to the inspired-to state is driven by the self-transcendence and attraction of a new consumption idea (Ryan and Deci, 2000), and the intrinsic motivation for autonomy and competence may be an internal driving force to implement new ideas regarding consumption (Böttger et al., 2017). Tourist inspiration is an extension of general inspiration in the field of tourism, and, thus, our findings showing the relationship between the inspired-by and inspired-to states are consistent with the extant literature on inspiration (Böttger et al., 2017).

The findings of this study show that the education experience positively and significantly influenced the inspired-by state of tourist inspiration. A strong beta (β) value for the relationship between education experience and inspired-by state of tourist inspiration reflects a strong impact of education experience on the inspired-by state as compared to the impacts of entertainment, esthetic, and escapism dimensions of the tourist experience. These findings are consistent with the work of He et al. (2021) where the scholars validated the relationship between education experience and tourist inspiration in the context of wellness tourism. The education experience improves tourist knowledge and skills through active learning during tourist visit to tourist destination (Oh et al., 2007). The acquisition of new knowledge and skills can enhance tourist cognition, broaden tourist horizons, and stimulate tourist imagination (Rudd et al., 2018) to help tourists discover new possibilities and ideas regarding consumption (Winterich et al., 2019). It is estimated that approximately 50% of the total inventions are inspired by new scientific knowledge (Callaert et al., 2014), and creativity is closely related to the application of new knowledge (Gurteen, 1998). Our findings support the works of Gurteen (1998) and Callaert et al. (2014).

The findings of this study also show that the entertainment experience significantly and positively influenced the inspired-by state of tourist inspiration. Compared with the other three dimensions of tourist experience, entertainment experience has a weak relationship with the inspired-by state of tourist experience due to the smallest path coefficient. The impact of the entertainment experience on tourist inspiration is investigated in this study which remained under-explored in the previous studies. Destination performances and activities inspire tourists by provoking tourists’ positive emotions and feelings of happiness, which are the prominent features of the entertainment experience (Oh et al., 2007). From the perspective of the broaden-and-build theory, positive emotions and feelings of happiness motivate tourist imagination, enhance tourist creativity, and promote tourist divergent thinking to help tourists discover new ideas regarding consumption (Fredrickson, 2001).

This study demonstrates that esthetics experience significantly and positively influenced the inspired-by state of tourist inspiration. Compared with education experience and escape experience, the path coefficient of the influence of esthetics experience on the inspired-by state is weak. These findings are consistent with the work of He et al. (2021). The esthetics experience reflects an individual’s cognitive response to the environment or events, which might be unique and different from others (Pine and Gilmore, 1998). Tourists’ appreciation of the environmental beauty and other objective matters of tourist destination reflects tourists’ inspiration and imagination (Böttger et al., 2017). The findings of this study provide robust support to the previous studies that demonstrated a strong relationship between imagination, i.e., the inspired-by state in this study, and esthetic experience (Brady, 1998; Joy and Sherry, 2003).

This study revealed that the escapism experience significantly and positively influenced the inspired-by state of tourist inspiration, which is also consistent with the work of He et al. (2021). However, compared with the other three dimensions of tourist experience, He et al. (2021) reported a strong path coefficient for the relationship between escapism experience and tourist inspiration, which differs from the findings of this study. The escapism experience refers to tourists’ escaping experiences from daily life and traveling to destinations to participate in specific activities (Oh et al., 2007). The previous literature has confirmed that escape experiences can be very memorable, pleasurable, and inspiring for tourists (Tom Dieck et al., 2018; Hwang and Lee, 2019). Different environments and lifestyles allow tourists to discover new possibilities and develop new ideas related to consumption with self-transcendence.

The findings of this study revealed a negative, however, weak, moderating impact of destination familiarity on the relationship between education experience and the inspired-by state of tourist inspiration. Nevertheless, the impact of entertainment, esthetics, and escapism dimensions of tourist experience on the inspired-by state of tourist inspiration was not negatively moderated by destination familiarity and the moderating impact remained comparatively weak due to co-efficient values near zero. Destination familiarity is described as tourist cognition and tourist knowledge and experience regarding tourist destination (Tan and Wu, 2016; Liu et al., 2018). As tourists become more familiar with destinations, fewer new possibilities are discovered, which reduces the probability of obtaining new ideas and exerts a little impact on tourist inspiration. Extant literature presents that familiarity negatively impacts imagination expansion (Sharman et al., 2005), which is consistent with the findings of this study. Entertainment, esthetics, and escapism dimensions of tourist experience are more related to tourist emotions and feelings and are less affected by cognition-based destination familiarity. Thus, destination familiarity exerts no moderating impact on the relationships among entertainment, esthetics, and escapism dimensions of tourist experience and tourist inspiration.

### Theoretical Contribution

This study makes several important theoretical contributions to the extant literature. First, from the general perspective of tourism, this study puts forth empirical evidence to demonstrate the impact of tourist experience, i.e., education, esthetics, entertainment, and escapism, on tourist inspiration, i.e., the inspired-by state, that provides a broader lens to DMOs to understand tourist cognitive responses for destination promotion, which previously remained context-specific and limited, such as wellness tourism (He et al., 2021) and international travel (Khoi et al., 2019). This study expands the critical understanding of the relationship between tourist experience and tourist inspiration by clarifying the influence of entertainment experience on tourist inspiration which remained inconsistent and mixed in the previous studies on conventional tourism and wellness tourism (He et al., 2021). Tourist inspiration reveals the psychological mechanism of tourist experience that promotes tourist purchase behavior. Tourist shopping or consumption behavior at a host destination is likely to be driven by tourist inspirations. Second, based on the transmission model of inspiration, which was proposed by Thrash et al. (2010) and Böttger et al. (2017) found that inspired-by state mediates the influence of marketing stimulus on inspired-to state. In the field of marketing, previous studies verified the significant impact of inspired-by state on the inspired-to state (Hinsch et al., 2020; Izogo et al., 2020). In the field of tourism, some empirical studies explored the antecedents and consequences of tourist inspiration (He et al., 2021; Khoi et al., 2021). However, the relationship between the inspired-by state and the inspired-to state, as the two components of tourist inspiration, remained deeply under-explored. This study explored tourist consumption inspiration and found that the inspired-by state of tourist inspiration can significantly and positively affect the inspired-to state in the context of tourism, thus, consolidating the theory of customer inspiration and extending the applicable boundaries of the general inspiration theory in the field of tourism. Third, He et al. (2021) found that education experience significantly affects tourist inspiration in the context of wellness tourism, and this relationship is positively moderated by openness to experience. Our findings show that the education experience can significantly and positively impact the inspired-by state of tourist inspiration and destination familiarity negatively moderates the relationship between education experience and the inspired-by state, which extends knowledge and understanding regarding the impact of tourist education experience on tourist inspiration for additional theoretical insights.

### Managerial Implications

Destination marketing organizations can design and arrange destination performances and events to enhance tourist experience, which may motivate tourist inspiration and increase destination sales revenue. Although the discussed four dimensions of tourist experiences significantly and positively affect tourist inspiration, DMOs need to combine their advantages to strategically position tourist experience of destinations. Based on such a positioning strategy, DMOs can plan related marketing activities to highlight specific tourist experiences in tourist destination to strengthen destination brand image. For example, the southern Sichuan Bamboo Sea is in Yibin City, Sichuan Province, China, and is a 4A-level natural scenic spot in China. To highlight the educational experience and aesthetic experience, the scenic spot has built the largest bamboo professional museum in China by combining the advantages of rich bamboo resources to show tourists the long history of Chinese bamboo culture and various bamboo crafts. Visiting the museum may inspire tourists to purchase bamboo craft products to increase scenic spot sales revenue. To highlight the escape and entertainment experiences, the scenic spot plans bamboo raft water experience activities. This inspires tourists to consume bamboo raft experience projects and, ultimately, to increase scenic spot sales revenue. Thus, tourist inspiration uncovered under the empirical lens of this study provides new strategic directions for DMOs.

For destinations characterized by education experience, DMOs need to consider the differences in tourist destination familiarity between revisiting and first-time tourists and adopt different marketing strategies for different types of tourists. For revisiting tourists, DMOs can use two marketing strategies to stop declining purchase motivation caused by the negative moderating effect of destination familiarity to maintain destination sales revenue. The first strategy is that using sales promotions and designing creative marketing campaigns may improve tourist purchase motivation and tourist inspiration, respectively. The second strategy is to increase tourist novelty experience during tourist visits to destinations to trigger tourist inspiration, which weakens the negative moderating effect of destination familiarity, with the help of continuous innovation in destination performances and events.

COVID-19 pandemic may negatively impact tourist willingness to visit familiar and unfamiliar tourist destinations, resulting in a huge impact on the global tourism industry due to the sharp drop in the number of tourists (Rasoolimanesh et al., 2021). The COVID-19 pandemic will likely impact tourist consumption patterns worldwide, such as the growing popularity of free and independent travel, luxury trips, and health and wellness tourism (Majeed and Ramkisson, 2020). The ongoing COVID-19 pandemic may have influenced people to reconsider their travel decisions for familiar and unfamiliar tourist destinations to avoid the risk of catching the COVID-19 virus during travel. From the perspective of wellness tourism experience, He et al. (2021) examined the influence of tourist experience on tourist inspiration during the COVID-19 pandemic, and the conclusions drawn by He et al. (2021) are consistent with the findings of this study, providing support to the applicability of the findings of this study during the ongoing context of the COVID-19 pandemic. Since our study was conducted before the COVID-19 pandemic, a comparison between the findings of our study and of He et al. (2021) open doors for DMOs for interesting takeouts of this study during and post the COVID-19 pandemic. However, tourist destinations need to reconsider their service designs and distribution channels to match tourists’ changing cognitive reactions and behavioral intention during and post the COVID-19 pandemic. Destinations can strengthen prevention and control measures at tourist attractions, such as controlling the density of tourists, increasing the requirement of negative nucleic acid tests, and frequent disinfection of the tourist attractions in densely populated areas, to reduce the perceived risk of catching the COVID-19 virus during tourist visit to tourist attractions. Improved prevention and control measures to combat the risk of catching the COVID-19 virus will increase tourist sense of security during visits to destination alongside enhancing tourist experience to trigger positive tourist inspiration.

### Limitations and Future Research Directions

This study explores the relationship between the dimensions of tourist experience and tourist inspiration. However, there are some limitations of this study that open doors for future research on the topic under investigation. There are many antecedents to tourist inspiration (Khoi et al., 2019; Winterich et al., 2019). However, the current study only explored four dimensions of tourist experience as antecedents to tourist inspiration. Future research can explore other factors that may influence tourist inspiration based on the traits of tourists and the characteristics of destinations for a profound understanding of tourist inspiration. Our study examined the moderating impact of destination familiarity on the relationship between tourist experience and tourist inspiration and found that destination familiarity has no moderating impact on the relationship between the proposed facets of tourist experience and tourist inspiration except for the relationship between education experience and inspired-by state of tourist inspiration. Future research can extend the scope of our work to investigate the impact of other moderators, such as national culture, on the relationship between tourist experience and tourist inspiration for additional insights.

Since this study explored the inspired-to state of tourist inspiration with a focus on purchase motivation, the proposed theoretical framework of the study can be extended to reflect how tourist inspiration affects tourist revisit intention, tourist wellbeing, and tourist intention to recommend a destination (Filep and Laing, 2019). Future research may also focus on other perspectives to examine tourist inspiration at different stages of travel. For example, how can tourist inspiration influence tourist intention to visit/revisit specific tourist destinations during the pre-travel planning phase? Since a growing number of tourists collect destination information online when making travel plans (Majeed and Ramkissoon, 2022), it will be worth exploring how DMOs can inspire tourists through online information and fuel tourist travel intention for a specific tourist destination.

## Data Availability Statement

All datasets generated for this study are included in the article/Supplementary Material, further inquiries can be directed to the corresponding author.

## Ethics Statement

This study was carried out in accordance with the recommendations of the Local Ethics Committee of Shenzhen. All the study participants provided written informed consent in accordance with the Declaration of Helsinki. The study protocol was approved by the Local Ethics Committee of Shenzhen University.

## Author Contributions

JX: conceptualization, conduct of the survey, data gathering, data analysis, revisions, development, and proofreading of the manuscript. ZZ: conceptualization and survey design. SM: revisions, development, and proofreading of the manuscript. RC: data analysis. NZ: survey design. All authors contributed to the article and approved the submitted version.

## Conflict of Interest

The authors declare that the research was conducted in the absence of any commercial or financial relationships that could be construed as a potential conflict of interest.

## Publisher’s Note

All claims expressed in this article are solely those of the authors and do not necessarily represent those of their affiliated organizations, or those of the publisher, the editors and the reviewers. Any product that may be evaluated in this article, or claim that may be made by its manufacturer, is not guaranteed or endorsed by the publisher.

## Footnotes

1
https://www.wjx.cn
