# Supplementary material for: Stimulating Tourist Inspiration by Tourist Experience: The Moderating Role of Destination Familiarity
Source: Front Psychol. 2022 Jul 1;13:895136. doi: 10.3389/fpsyg.2022.895136 (PMC9284030; doi:10.3389/fpsyg.2022.895136)
Supplement: Supplementary file 1 [file Table_1.DOCX]

**Appendix 1**

**Questionnaire survey scale items**

Stimulating Tourist Inspiration by Tourist Experience: The Moderating Role of Destination Familiarity

Please choose one option that best describes your thought.

|  | **Strongly disagree (1)** | **Partially disagree (2)** | **Disagree (3)** | **Neutral (4)** | **Agree**  **(5)** | **Partially agree (6)** | **Strongly agree (7)** |
| --- | --- | --- | --- | --- | --- | --- | --- |
| **Tourist experience** |  |  |  |  |  |  |  |
| **Education experience** |  |  |  |  |  |  |  |
| This tour has expanded my knowledge. |  |  |  |  |  |  |  |
| This tour allowed me to learn a lot. |  |  |  |  |  |  |  |
| This tour inspired my curiosity about learning new things. |  |  |  |  |  |  |  |
| This tour was a learning experience. |  |  |  |  |  |  |  |
| **Esthetics** **experience** |  |  |  |  |  |  |  |
| This tour made me feel harmony. |  |  |  |  |  |  |  |
| This tour was extremely pleasant. |  |  |  |  |  |  |  |
| The surroundings of tourist destination attractions were pretty bland. |  |  |  |  |  |  |  |
| The destination environment was very attractive. |  |  |  |  |  |  |  |
| **Entertainment** **experience** |  |  |  |  |  |  |  |
| Activities at tourist destinations were amusing to watch. |  |  |  |  |  |  |  |
| The performance at tourist destination was captivating to watch. |  |  |  |  |  |  |  |
| I enjoyed watching the performance at tourist destination. |  |  |  |  |  |  |  |
| Activities at tourist destination were fun to watch. |  |  |  |  |  |  |  |
| **Escapism experience** |  |  |  |  |  |  |  |
| I feel different on this tour. |  |  |  |  |  |  |  |
| This trip made me feel like living in another space and time. |  |  |  |  |  |  |  |
| This trip made me imagine being another self. |  |  |  |  |  |  |  |
| This trip allowed me to escape from reality. |  |  |  |  |  |  |  |
| **Inspired-by** |  |  |  |  |  |  |  |
| This tour activated my imagination. |  |  |  |  |  |  |  |
| A new idea on this tour caught my interest. |  |  |  |  |  |  |  |
| This trip unexpectedly and spontaneously gave me new ideas. |  |  |  |  |  |  |  |
| This trip has broadened my horizons. |  |  |  |  |  |  |  |
| This tour made me discover something new. |  |  |  |  |  |  |  |
| **Inspired-to** |  |  |  |  |  |  |  |
| This tour inspired me to buy something related to tourist attractions. |  |  |  |  |  |  |  |
| This trip gave me a desire to buy something related to tourist attractions. |  |  |  |  |  |  |  |
| This tour increased my interest in buying something related to tourist attractions. |  |  |  |  |  |  |  |
| This trip motivated me to buy something related to tourist attractions. |  |  |  |  |  |  |  |
| This trip gave me the urge to buy something related to tourist attractions. |  |  |  |  |  |  |  |
| **Destination familiarity** |  |  |  |  |  |  |  |
| Before going to tourist attractions, I had more knowledge of tourist attractions than average people. |  |  |  |  |  |  |  |
| Before going to tourist attractions, I had more knowledge of tourist attractions than my friends. |  |  |  |  |  |  |  |
| Before going to tourist attractions, I had more knowledge of tourist attractions than people who travel frequently. |  |  |  |  |  |  |  |

**Thank you for participating!**
