# Supplementary material for: Stimulating Tourist Inspiration by Tourist Experience: The Moderating Role of Destination Familiarity
Source: Front Psychol. 2022 Jul 1;13:895136. doi: 10.3389/fpsyg.2022.895136 (PMC9284030; doi:10.3389/fpsyg.2022.895136)
Supplement: Supplementary file 2 [file Table_2.DOCX]

**Appendix 2**

**Table** Results of mediation impact

| Path relationship | Indirect effect/*β* value | *t*-value | LLCI | ULCI |
| --- | --- | --- | --- | --- |
| Education -> Inspired-by -> Inspired-to | 0.161^***^ | 7.689 | 0.120 | 0.203 |
| Entertainment -> Inspired-by -> Inspired-to | 0.079^***^ | 4.090 | 0.044 | 0.122 |
| Escapism -> Inspired-by -> Inspired-to | 0.100^***^ | 5.748 | 0.068 | 0.135 |
| Esthetics -> Inspired-by -> Inspired-to | 0.094^***^ | 4.614 | 0.057 | 0.137 |
| Note: LLCI: lower limit of confidence interval, ULCI: upper limit of confidence interval; ****p* < 0.001. | | | | |
